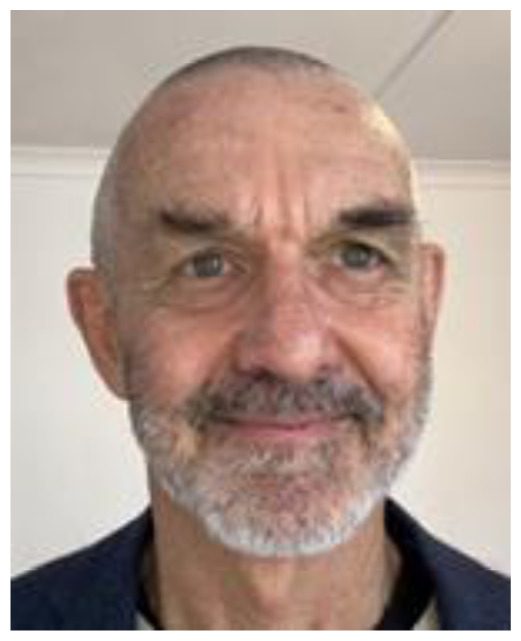# Entering the era of artificial intelligence (AI) in publishing

**DOI:** 10.17159/2078-516X/2023/v35i1a15511

**Published:** 2023-01-31

**Authors:** Mike Lambert

The South African Journal of Sports Medicine (SAJSM) published 36 papers in 2022. Fifty-eight percent of the submitted papers were not accepted. The year was spent preparing the published manuscripts from 2019 onwards for the National Library of Medicine’s PubMed database. It is a technically difficult task, so it was outsourced to a company in the USA which specializes in file conversions. They provided an excellent service so the SAJSM will continue to use them in the future. File conversions adds another dimension to managing the journal because we have to cover the costs associated with this service. The South African Sports Medicine Association (SASMA) has been covering the costs of the journal since 1983, but the annual budget of the journal now exceeds the amount they are able to contribute. Commercial partners who could contribute to the running costs of the journal were sought, but this was not successful. This has necessitated a change in the publishing model of the journal to include publication costs for the authors. A “sweet spot” cost has been calculated that will be affordable for the authors and meaningful for the journal’s budget. Authors who are SASMA members will get a reduced rate. The SAJSM is also accepting adverts which will be displayed on the opening page of the website. This new publishing model will enable the journal to grow and be self-sustaining. It will also reduce the financial burden on SASMA which has supported the journal for four decades.

The SAJSM has identified other challenges. For example, it is increasingly difficult to get good quality reviewers. Reviewing is an altruistic job because there is no external recognition or payment. With a double-blind review, the identity of the reviewer is only known to the editorial staff. There is no reward for the reviewer, apart from the good feeling associated with making a contribution to science and the development of knowledge. Also, the process of peer review is not perfect. Personal biases may creep into the review and the original thoughts of the authors may get blocked or redirected by the reviewer. This is unfortunate because it contaminates the system designed to maintain the progression of knowledge. A better system of checking the quality of manuscripts before publication has not yet been devised, so this imperfect system has to be used. It is a problem all journals encounter. The SAJSM has addressed the problem by offering training courses for potential reviewers. These courses teach the principles of reviewing and work on the premise that the principles of reviewing a paper can be applied to writing a manuscript. In other words, reviewers with good reviewing skills usually have good writing skills. The SAJSM is also discussing how reviewers working in a health professional environment can get continuing professional development (CPD) points for completing a review. Another incentive for reviewing, particularly for reviewers working in an academic environment, is to subscribe to Publons. This is a website that tracks and verifies peer review reports for academics. Publons has gained popularity and now has 3 000 000 subscribed reviewers. In 2017 Clarivate bought Publons and incorporated it into the Web of Science. Publons produces a comprehensive report of all the reviews the subscribers have completed. Reports can be downloaded and used to boost an academic CV. The SAJSM is planning to automate the process of linking the reviewer’s report to Publons as an incentive to reviewers. These are just some of the strategies which will be implemented to reduce the burden of reviewing.

The next challenge the SAJSM faces is to identify non-authentic work which is submitted for review. To guard against this every paper submitted to the journal is submitted to iThenticate. This software checks plagiarism. In 2022 three papers were rejected before review because significant parts of them had been published previously. The challenge of detecting plagiarism is compounded by the public’s access to ChatGPT, AI software released to the public in November 2022. ChatGPT generates original text based on written prompts. Text generated by ChatGPT is not detected by iThenticate. Stories abound about the miraculous things the software can do. For example, ChatGPT can write well-constructed essays, personalised bedtime stories for children, poetry and lyrics for songs. Perhaps the most publicised feat was the software getting a grade (B) on a Wharton Business School test. Microsoft announced in January 2023 that it had made a ~$10 billion investment into the company OpenAI which developed ChatGPT. Massive investments in technology are only made in exceptional circumstances when it is clear the product will change the trajectory of the development of society. ChatGPT is such a case. There is no doubt that this software is going to have a major impact on education. There is concern among educators that students will rely on ChatGPT for much of their work and many of them will. Many scientists will also use Chat GPT as a lazy shortcut. If the software is used as a crutch it will hinder the development of critical thinking and problem-solving skills. These skills are a prerequisite for many jobs, and scientists need these skills to answer questions and communicate the results. Students and scientists without these skills will lag behind and become less competitive. A counterargument is that if ChatGPT is used appropriately it can be a tool which assists in developing these skills. An analogy would be how calculators serve mathematics. When calculators became available to scholars there was concern they were going to be a threat to teaching mathematics. However, the discipline adapted and using calculators has become integral to teaching and applying mathematics. The same logic applies to scientists who try and use the ChatGPT software as a quick fix to their writing and research. If the software is not used in an innovative way, the scientists will drift towards mediocrity because their work will lose the creative edge that can only come from original critical thinking.

It is too early to predict how this new software will affect us. There are compelling arguments for and against it. Our challenge is to remain alert for positive and negative effects and adjust accordingly. Failing to do so will have many unintended consequences.[Fig f1-2078-516x-35-v35i1a15511]

**Figure f1-2078-516x-35-v35i1a15511:**